# Significance of tonsillectomy combined with steroid pulse therapy for IgA nephropathy with mild proteinuria

**DOI:** 10.1007/s10157-015-1138-7

**Published:** 2015-06-30

**Authors:** Hiroyuki Komatsu, Yuji Sato, Tetsu Miyamoto, Masahito Tamura, Takeshi Nakata, Tadashi Tomo, Tomoya Nishino, Masanobu Miyazaki, Shouichi Fujimoto

**Affiliations:** First Department of Internal Medicine, University of Miyazaki Hospital, 5200 Kihara, Kiyotake, Miyazaki, 889-1692 Japan; Second Department of Internal Medicine, University of Occupational and Environmental Health School of Medicine, Kitakyushu, Japan; Department of Endocrinology Metabolism, Rheumatology and Nephrology, Faculty of Medicine, Oita University, Oita, Japan; Blood Purification Center, Oita University of Hospital, Oita, Japan; Second Department of Internal Medicine, Nagasaki University School of Medicine, Nagasaki, Japan; Department of Hemovascular Medicine and Artificial Organs, Miyazaki University School of Medicine, Miyazaki, Japan

**Keywords:** IgA nephropathy, Glomerulonephritis, Tonsillectomy, Steroid pulse therapy, Remission

## Abstract

**Background:**

Medical intervention for patients with IgA nephropathy and mild proteinuria (<1.0 g/day) is controversial, and the effectiveness of tonsillectomy plus steroid pulse therapy (TSP) for such patients remains obscure.

**Methods:**

Among 323 patients in our multicenter cohort study, 79 who had mild proteinuria (0.4–1.0 g/day) at diagnosis were eligible to participate in this study. We compared the clinicopathological findings at diagnosis, a decline in renal function defined as a 50 or 100 % increase in serum creatinine (sCr) and clinical remission (CR) defined as the disappearance of hematuria and proteinuria (<0.3 g/day) among groups given TSP (*n* = 46), steroid therapy (ST) (*n* = 9), and non-ST (*n* = 24). Factors contributing to CR were also evaluated using multivariate analysis.

**Results:**

Background factors at diagnosis including age, ratio (%) of patients with hypertension, sCr, proteinuria, and histological severity did not significantly differ among the groups. Only two patients each in the TSP (4.3 %) and non-ST (8.3 %) groups achieved a 50 % increase in sCr during a mean follow–up period of 4.7 years. At the final observation, 71.7, 44.4, and 41.7 % of patients in the TSP, ST, and non-ST groups, respectively, achieved CR (*p* = 0.032). Cox proportional hazards models revealed that TSP led to CR more effectively than non-TSP by a factor of about threefold (hazard ratio, 2.74; *p* = 0.008).

**Conclusion:**

TSP therapy has potential for inducing CR in patients with IgAN and mild proteinuria (<1.0 g/day).

## Introduction

Immunoglobulin A nephropathy (IgAN) is prominently associated worldwide with kidney disease as it occurs at high frequency in patients with glomerulonephritis [1, 2]. It is also the leading cause of glomerulonephritis in the Japan-Renal Biopsy Registry database, where it accounts for 30 % of all registered patients [3]. Although it has a worse renal prognosis with 30 to 40 % of affected patients reaching end-stage kidney disease (ESKD) within 20 years [4], a disease-specific treatment modality has not yet been established [5].

The clinical practice guidelines for glomerulonephritis published by Kidney Disease: Improving Global Outcomes (KDIGO) suggest a six-month cause of corticosteroid therapy for patients with IgAN who have persistent proteinuria >1.0 g/day despite three to six months of optimized supportive care, and glomerular filtration rates (GFR) >50 mL/min/1.73 m^2^ [6]. On the other hand, the propriety of treatment intervention for patients with IgAN who have mild proteinuria (<1.0 g/day) remains controversial [6, 7]. Some observational studies emphasize that even mild proteinuria might be a risk factor for a poor renal prognosis [8–12].

Some recent Japanese studies have examined the effects of tonsillectomy plus steroid pulse (TSP) therapy on urinary findings [13–17]. A multicenter randomized controlled trial indicated that TSP therapy exerts antiproteinuric effects, but one of the inclusion criteria comprised proteinuria of 1.0–3.5 g/day [17].

Therefore, we conducted a new cohort study to clarify whether TSP for patients with IgAN and mild proteinuria (<1.0 g/day) can ameliorate urinary parameters and renal function compared with non-TSP therapy.

## Methods

### Study design and participant selection

The Ethics Committees at each of the University of Occupational and Environment Health (approval number, H23-89), University of Nagasaki (number, 11102452-2), University of Oita (number, 496) and University of Miyazaki (number, 874) hospitals approved this multi-center retrospective cohort study.

The inclusion criteria comprised the patients who were initially diagnosed with IgAN by renal biopsy at the four institutions between September 1, 2000 and August 31, 2010, age, 16–60 years and followed-up for at least 1 year. Exclusion criteria comprised renal lesions caused by systemic diseases such as Henoch–Schönlein purpura nephritis, systemic lupus erythematosus, and liver cirrhosis.

Among 323 patients registered based on the initial criteria, we selected 79 with proteinuria of 0.4–1.0 g/day at diagnosis. We assigned them into groups according to treatment modalities (TSP, *n* = 46; steroid therapy, ST, *n* = 9; non-ST, *n* = 24). The clinicopathological findings at diagnosis, the effects of treatment on urinary findings and renal function were compared among the groups during a follow-up period of 4.70 ± 2.73 years.

### Evaluation of clinical findings

All basic information about age, sex, blood pressure (BP), urinary dip-stick findings and sediment, amount of 24-h proteinuria, urinary protein-creatinine ratio (UP/UCr), estimated GFR (mL/min/1.73 m^2^) and serological findings of creatinine (Cr), albumin, total cholesterol, triglyceride, C-reactive protein, IgA, and C3 at diagnosis, and initial treatment was collected from medical records. Information about renal function (sCr, eGFR) and urinary findings during follow-up and final observation was assessed and registered by investigators at each institution. Hypertension was defined as systolic BP >140 mmHg and/or diastolic BP >90 mmHg or medication with antihypertensive drugs before diagnosis. The qualitative findings of urinary protein (UP) and urinary occult blood (UOB) were each scored as follows: − and ± as 0; 1+ as 1; 2+ as 2; 3+ as 3.

### Evaluation of pathological findings

We assessed histological lesions from all patients according to the guidelines of the Special Society Group (IgAN) on Progressive Glomerular Disease in Japan.

The second version of the guidelines [18] separates patients with IgAN based on severity into Grades 1–4 as follows:

*Grade 1* Slight mesangial cell proliferation and increased matrix; absence of glomerulosclerosis, crescent formation, or adhesion to Bowman’s capsule; no prominent changes in interstitium, renal tubuli, or blood vessels.

*Grade 2* Slight mesangial cell proliferation and increased matrix; glomerulosclerosis, crescent formation, or adhesion to Bowman’s capsule in <10 % of all biopsied glomeruli; interstitial and vascular findings identical to those of grade 1.

*Grade 3* Moderate, diffuse mesangial cell proliferation and increased glomerulosclerosis, crescent formation, or adhesion to Bowman’s capsule in 10 to 30 % of all biopsied glomeruli; slight cellular infiltration in interstitium, except around some sclerosed glomeruli, slight tubular atrophy, and mild vascular sclerosis.

*Grade 4* Severe, diffuse mesangial cell proliferation and increased matrix; glomerulosclerosis, crescent formation, or adhesion to Bowman’s capsule in >30 % of all biopsied glomeruli. When sites of sclerosis are totaled and converted to global sclerosis, the sclerosis rate includes >50 % of glomeruli; some glomeruli also show compensatory hypertrophy, interstitial cellular infiltration, tubular atrophy and fibrosis; hyperplasia or degeneration evident in some intrarenal arteriolar walls.

The third version of the guidelines [19] also semi-quantitatively classifies severity into four grades based on glomerular lesions such as global/segmental glomerulosclerosis and cellular/fibrocellular/fibrous crescents. The ratios (%) of damaged glomeruli with at least one of the above findings among all observed glomeruli are histologically graded as H-grades of I, II, III, and IV (0–24.9, 25–44.9, 50–74.9, and >75 %, respectively). The damaged glomeruli were also categorized as acute (cellular and fibrocellular crescents) lesion (A), chronic (global/segmental glomerulosclerosis and fibrous crescents) lesion (C), and acute and chronic lesions (A/C), indicating to the disease stage.

### Definition of remission and study outcomes

The primary outcome was clinical remission (CR) defined as the disappearance of hematuria and proteinuria. The disappearance of hematuria was defined as at least two consecutive findings of <5/HPF of red blood cells in sediment or (−) ~ (±) in the dip-stick test. The disappearance of proteinuria was also defined as at least two consecutive findings of <0.3 g/day of protein in 24-h urine, a UP/UCr ratio of <0.3 in spot urine or (−) ~ (±) in the dip-stick test. The secondary outcome was a decline in renal function of 50 % or 100 % increase in sCr from baseline or ESKD with renal replacement therapy.

### Statistical analysis

All continuous variables are presented as mean ± standard deviation (SD). Clinical parameters of the three groups were compared using a single-factor analysis of variance (ANOVA) for normally distributed continuous variables or the Kruskal–Wallis test for non-normally distributed continued variables. Differences in proportions were evaluated using the *χ*^2^ independent test. Histological grades among three groups were compared using Pearson’s *χ*^2^ test. Cumulative probabilities of remission for each urinary finding were analyzed using the Kaplan–Meier method, and differences in curves were compared using the log-rank test. The impact of multiple covariates for the rate of CR was assessed using the Cox proportional hazards model. All independent variables used in the multivariate analyses were either categorical (coded as 0/1) or quantitative. Treatment with TSP and renin-angiotensin system inhibitors (RAS-I) were regarded as categorical variables. Age, systolic BP, UP concentration, sCr concentration, and histological grade were regarded as quantitative variables. The results of the multivariate analysis are expressed as hazard ratios (HR) meaning ratios for CR with a 95 % confidence interval (CI). A *p* value of <0.05 was considered significant for all data, which were statistically analyzed using IBM SPSS Advance Statistical Version 22.0.

## Results

### Clinicopathological findings at diagnosis and initial treatment

Table 1 shows the background factors at diagnosis of the groups. Levels of systolic and diastolic BP and the ratios of patients with hypertension did not differ among the groups. The 24-h proteinuria values were essentially comparable with a mean range of 0.63–0.69 g/day in all groups. Renal function determined as sCr and eGFR values was apparently within the normal range in all groups, and did not significantly differ. Histological severity estimated using the second and third versions of the grading systems also did not significantly differ, although the ratios of H-Grade I group with acute (A) and acute/chronic (A/C) lesions were higher in the TSP, than in the other two groups.

**Table 1:** Baseline characteristics of groups before treatment (*n* = 79)

	Groups			
	TSP (*n* = 46)	ST (*n* = 9)	Non-ST (*n* = 24)	p*
Age (year)	32.1 ± 12.9	34.0 ± 13.6	38.6 ± 17.4	0.204
Sex (M/F)	15/31	5/4	11/13	0.416
Systolic BP (mmHg)	124.1 ± 15.4	128.7 ± 13.8	125.0 ± 17.7	0.731
Diastolic BP (mmHg)	74.8 ± 13.3	77.0 ± 11.2	72.4 ± 10.8	0.583
Patients with >140/90 mmHg (*n*)	9 (19.6 %)	2 (22.2 %)	4 (16.7 %)	0.925
UOB score	2.43 ± 0.69	1.89 ± 0.93	2.13 ± 1.15	0.146
Proteinuria (g/day)	0.63 ± 0.19	0.69 ± 0.20	0.66 ± 0.20	0.607
Serum creatinine (mg/dL)	0.75 ± 0.25	0.86 ± 0.23	0.85 ± 0.32	0.233
Estimated GFR (mL/min/1.73 m^2^)	92.5 ± 28.5	77.6 ± 14.1	79.9 ± 28.8	0.109
Serum albumin (g/dL)	4.17 ± 0.34	4.13 ± 0.58	4.14 ± 0.48	0.936
Serum total cholesterol (mg/dL)	197.0 ± 30.0	200.9 ± 37.0	207.7 ± 50.8	0.543
Serum IgA (mg/dL)	326.0 ± 125.9	343.4 ± 115.1	333.5 ± 139.0	0.923
Serum C3 (mg/dL)	107.5 ± 19.1	108.1 ± 26.5	107.4 ± 25.3	0.996
Histological severity (2nd version)				0.312
1	6 (13.0 %)	0 (0.0 %)	7 (29.2 %)	
2	11 (23.9 %)	1 (11.1 %)	5 (20.8 %)	
3	22 (47.8 %)	7 (77.8 %)	9 (37.5 %)	
4	7 (15.2 %)	1 (11.1 %)	3 (12.8 %)	
Histological severity (3rd version)				0.170
H-Grade I	28 (60.9 %)	3 (33.3 %)	14 (58.3 %)	
(A, A/C, C)	(17, 11, 0)	(2, 0, 1)	(8, 5, 1)	
H-Grade II	14 (30.4 %)	4 (44.4 %)	8 (33.3 %)	
(A, A/C, C)	(3, 8, 3)	(0, 2, 2)	(2, 3, 3)	
H-Grade III	4 (8.7 %)	1 (11.1 %)	2 (8.3 %)	
(A, A/C, C)	(0, 4, 0)	(0, 1, 0)	(1, 0, 1)	
H-Grade IV	0 (0.0 %)	1 (11.1 %)	0 (0.0 %)	
(A, A/C, C)		(0, 0, 1)		

*HT* hypertenstion, *A* acute lesions (cellular crescent, fibrocellular crescent), *C* chronic lesions (global/segmental sclerosis, fibrous crescent), *A*/*C* acute and chronic lesions

*ANOVA, Chi square independent test or Pearson’s Chi square test

The TSP group tended to undergo more courses of initial of steroid pulse therapy than the ST group but the difference did not reach statistical significance (*p* = 0.156, Table 2). Almost all patients received intravenous administration of methylprednisolone 0.5 g/day for 3 consecutive days as one course of pulse therapy. Initial dose of oral corticosteroid after steroid pulse therapy was 20–30 mg/day and continued for about 12–18 months. About 30 % of the patients in the non-ST group were treated by tonsillectomy without ST. The ratio of patients who had been treated with RAS-I was higher in the ST and non-ST, than in the TSP groups (77.8 vs. 66.7 vs. 47.8 %).

**Table 2:** Comparison of initial treatment among groups (*n* = 79)

	Groups			
	TSP (*n* = 46)	ST (*n* = 9)	Non-ST (*n* = 24)	p*
Steroid pulse therapy (mPSL 0.5 g/day × 3 days)				0.156
One course	18 (39.1 %)	3 (33.3 %)	–	
Two courses	18 (39.1 %)	1 (11.1 %)	–	
Three courses	10 (21.8 %)	1 (11.1 %)	–	
Oral steroid therapy (Initial dose of prednisolone)				0.279
<20 mg/day	1 (2.2 %)	0 (0.0 %)	–	
20 mg/day	13 (28.3 %)	5 (55.6 %)	–	
25 mg/day	7 (15.2 %)	1 (11.1 %)	–	
30 mg/day	25 (54.3 %)	3 (33.3 %)	–	
Tonsillectomy	46 (100 %)	0 (0.0 %)	7 (29.2 %)	<0.001*
RAS-I	22 (47.8 %)	7 (77.8 %)	16 (66.7 %)	0.183
Anti-platelet agents	42 (91.3 %)	7 (77.8 %)	17 (70.8 %)	0.080
Immunosuppressive agents	0 (0.0 %)	0 (0.0 %)	1 (4.2 %)	0.772

*mPSL* methilprednisolone, *RAS-I* renin-angiotensin system inhibitors

* ANOVA, Chi square independent test or Pearson’s Chi square test

### Remission rates and renal function at final observation

**Fig. 1:**
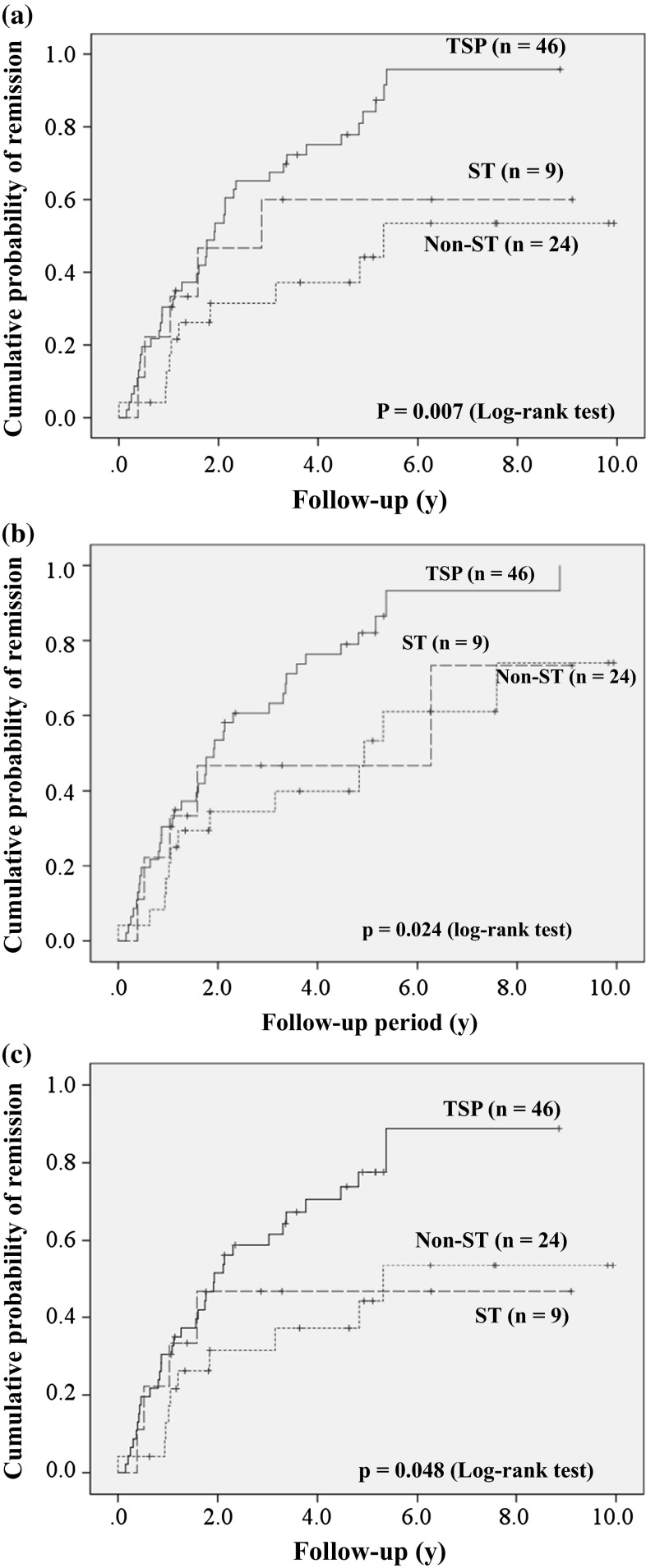
Comparison of cumulative probabilities of remission for urinary findings according to treatment modality. Remission of proteinuria (**a**), hematuria, (**b**) and clinical remission (**c**). Statistical differences in curves were compared using log-rank tests. *ST* steroid therapy, *TSP* tonsillectomy combined with steroid pulse therapy

**Fig. 2:**
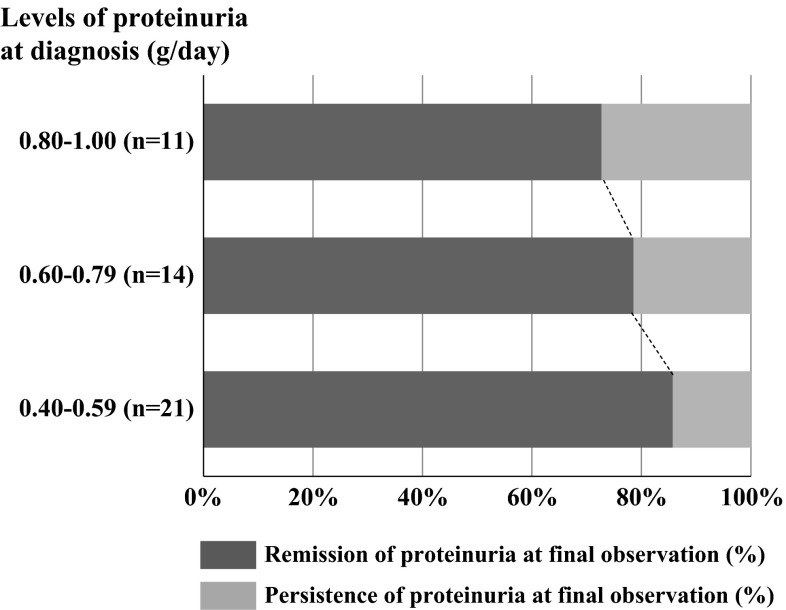
Relationship between the remission of proteinuria at final observation and levels of urinary protein at diagnosis. The each ratio of remission of proteinuria was 85.7 % in 0.40–0.59 g/day, 78.6 % in 0.60–0.79 g/day, 72.7 % in 0.80–1.0 g/day

Table 3 shows the remission rates, urinary findings, and renal function at the final observation. The ratio of patients with short duration of nephropathy (<3 years from the onset of urinary abnormality to the initiation of treatment intervention) was higher in TSP and ST groups than in non-ST groups (63.0 vs. 55.6 vs. 25.0 %, *p* = 0.009). During a follow-up period of 4.3–5.3 years, the mean amount of 24-h proteinuria fell in the TSP group (0.63 ± 0.19 to 0.30 ± 0.63 g/day), but increased in the ST group and non-ST groups (0.69 ± 0.20 to 0.89 ± 1.17 and 0.66 ± 0.20 to 1.17 ± 1.16 g/day, respectively). The ratio of proteinuria and hematuria remission was significantly higher in TSP, than in the ST and non-ST groups (proteinuria: 80.4 vs. 55.6 and 41.7 %; *p* = 0.001; hematuria: 82.6 vs. 55.6 and 54.2 %; *p* = 0.025). Furthermore, the ratio of CR in the TSP group was significantly higher than that in other two groups (71.7 vs. 44.4 vs. 41.7 %, *p* = 0.032). Kaplan–Meier analysis also uncovered differences in the cumulative probability of remission for the urinary findings (proteinuria, *p* = 0.007; hematuria, *p* = 0.024; CR, *p* = 0.048; log-rank test; Fig. 1). Treatment efficacy of TSP on the ratio of disappearance of proteinuria at final observation tended to depend on the amount of urinary protein at diagnosis, although the difference was not statistically significant (85.7 % in 0.40–0.59 g/day, 78.6 % in 0.60–0.79 g/day, 72.7 % in 0.80–1.0 g/day; *p* > 0.05, Fig. 2).

**Table 3:** Comparison of clinical findings at final observation (*n* = 79)

	Group			
	TSP (*n* = 46)	ST (*n* = 9)	Non-ST (*n* = 24)	p*
Duration of nephropathy^a^				0.022*
<1 year	19 (41.3 %)	1 (11.1 %)	5 (20.8 %)	
1–3 years	10 (21.7 %)	4 (44.4 %)	1 (4.2 %)	
3–5 years	8 (17.4 %)	1 (11.1 %)	7 (29.2 %)	
>5 years	9 (19.6 %)	3 (33.3 %)	11 (45.8 %)	
Observation period (year)	4.34 ± 2.25	5.29 ± 3.81	5.16 ± 3.12	0.398
Proteinuria (g/day)	0.30 ± 0.63	0.89 ± 1.17	1.17 ± 1.16	0.001*
Remission of proteinuria (*n*, %)	37 (80.4 %)	5 (55.6 %)	10 (41.7 %)	0.001*
Remission of hematuria (*n*, %)	38 (82.6 %)	5 (55.6 %)	13 (54.2 %)	0.025*
Clinical remission (*n*, %)	33 (71.7 %)	4 (44.4 %)	10 (41.7 %)	0.032*
Serum creatinine (mg/dL)	0.77 ± 0.20	0.84 ± 0.18	1.26 ± 1.74	0.139
Estimated GFR (mL/min/1.73 m^2^)	83.8 ± 25.8	75.6 ± 13.0	71.1 ± 33.3	0.183
Patients with 50 % increased sCr (*n*, %)	2 (4.3 %)	0 (0.0 %)	2 (8.3 %)	0.878
Patients with 100 % increased sCr or ESKD (*n*, %)	0 (0.0 %)	0 (0.0 %)	1 (4.2 %)	0.912
Adverse effect by the treatment				
Infection^b^	1 (2.2 %)	0 (0.0 %)	0 (0.0 %)	
Hyperglycemia	6 (13.0 %)	1 (11.1 %)	0 (0.0 %)	
Depression	1 (2.2 %)	0 (0.0 %)	0 (0.0 %)	
Palpitation	3 (6.5 %)	1 (11.1 %)	0 (0.0 %)	
Sleep disorder	8 (17.4 %)	2 (22.2 %)	1 (4.2 %)	

* ANOVA or Pearson’s Chi square test

^a^Period from the first manifestation of urinary abnormalities to the initiation of treatment intervention

^b^A case with esophageal candida

Renal function estimated as mean sCr and eGFR values at final observation did not significantly differ among the groups. Only two (4.3 %) patients in the TSP group and two (8.3 %) in the non-ST group reached a 50 % increase in sCr from baseline. One patient (4.2 %) in the non-ST group eventually progressed to ESKD (Table 3).

Adverse effects which may be mainly caused by steroid drugs during the initial treatment occurred especially in TSP group (Table 3). One patient (2.2 %) had an onset of esophageal candida. Three (6.5 %) of six patients (13.0 %) with hyperglycemia transiently needed oral antidiabetic agents. Three patients (6.5 %) complained about transient palpitation during steroid pulse therapy.

### Analysis of factors contributing to clinical remission

The Cox proportional hazards model was applied to evaluate the effects of the clinicopathological findings and modality of initial treatment on CR. The known major risk factors for IgAN progression as well as TSP and RAS-I were selected as imperative independent variables in the model (Table 4). A higher UP value before treatment was more likely to be a resistance factor for CR, although statistical significance was not proven in the univariate analysis. By contrast, TSP was a favorable factor to induce CR in the univariate and multivariate analyses. The combined therapy was about threefold more effective than the non-combined therapies in causing CR (HR 2.74; 95 % CI 1.30–5.77, *p* = 0.008).

**Table 4:** Multivariate analysis of factors contributing to clinical remission (*n* = 79)

Variable	Univariate analysis			Multivariate analysis		
	Hazard ratio	95 % CI	*p*	Hazard ratio	95 % CI	*p*
Age (/10 years)	0.926	(0.741–1.157)	0.499	0.897	(0.652–1.235)	0.504
Systolic BP (/10 mmHg)	0.991	(0.833–1.180)	0.922	0.952	(0.763–1.190)	0.667
Urinary protein (/0.1 g/day)	0.188	(0.034–1.049)	0.057	0.247	(0.041–1.485)	0.127
Estimated GFR (/10 mL/min/1.73 m^2^)	1.013	(0.900–1.140)	0.827	0.917	(0.779–1.080)	0.299
Histological severity (3^rd^) (/grade)	1.062	(0.706–1.598)	0.773	1.206	(0.747–1.946)	0.444
TSP versus non-TSP therapies	2.730	(1.346–5.539)	0.005*	2.740	(1.302–5.768)	0.008*
Presence versus absence of RAS-I	0.683	(0.357–1.305)	0.248	0.855	(0.416–1.760)	0.672

* Statistically significant

## Discussion

Whether or not patients with IgAN accompanied by mild proteinuria and normal renal function should be treated imposes a considerable dilemma, since such patients have not received much focus [6, 20]. Patients with “mild” IgAN might be able to retain long-term renal function with or without spontaneous remission [21]. On the other hand, some studies emphasize that 30 to 40 % of patients with minimal proteinuria (<0.4 g/day) and normal renal function eventually progress to overt disease stages indicated by overt signs such as increased proteinuria (>1.0 g/day), impaired renal function, and onset of hypertension [8–10]. Coppo also refers to the “legacy effect”, which is the memory of a treatment that produces benefits long after the regimen is concluded, especially at the early stage of IgAN [7]. The present study found that the combination of tonsillectomy and steroid pulse therapy at the early stage of IgAN was beneficial because the ratio (%) of CR was significantly higher for patients treated with the combination therapy (71.7 %) than steroid monotherapy (44.4 %), other therapies (41.7 %), the comparable administration of RAS-I, or spontaneous remission (presumed in 10 % to 30 % of patients).

Combination TSP therapy with or without RAS-I and antiplatelet agents became the first-choice treatment modality for IgAN according to a nationwide survey between 2004 and 2008 in Japan [22, 23]. Some recent Japanese studies have also investigated the effects of TSP therapy on clinical outcomes, especially upon urinary remission [13–17]. The KDIGO clinical practice guidelines for glomerulonephritis recommends a 6-month course of corticosteroid therapy for patients with persistent proteinuria >1 g/day despite 3–6 months of optimized supportive care and GFR >50 mL/min/1.73 m^2^. Meanwhile, tonsillectomy is not recommended except for patients with gross hematuria and tonsillitis [6]. Indeed, the level of evidence based on the quality of the study design considerably differs between corticosteroid therapy and tonsillectomy. By contrast, evaluations of TSP therapy itself are insufficient, since the concept that tonsillectomy can be combined with steroid pulse therapy has not been established.

Hotta et al. initially proposed a mechanism of action for combination therapy during the onset and progression of IgAN [24]. Chronic antigenic stimulation of the tonsillar mucosa causes the production of aberrantly glycosylated IgA1 mainly in bone marrow via the “mucosa-bone marrow axis”, and the deposition of aberrantly glycosylated IgA1 within the mesangial area increases glomerular damage. Tonsillectomy might act upstream of the mechanism by eliminating antigenic stimuli from the tonsillar mucosa, whereas steroid pulse therapy acts downstream by suppressing the abnormal immune response in the bone marrow, which leads to subsequent inflammation in renal glomeruli. Thus, intervention against both pathogenic streams might have a synergetic therapeutic effect on IgAN. Moreover, the larger number of courses of steroid pulse therapy might lead to the increase of the patients with CR.

Basic research has generated some new insights into the relationship between the pathogenesis of IgAN and TSP therapy [25–27]. Responses to TSP therapy are better for patients who express high levels than low levels of tonsillar Toll-like receptor (TLR) 9, a pathogen recognition molecule that discriminates pathogens from self [25]. Serum levels of galactose-deficient IgA1 and IgA/IgG immune complexes are significantly decreased in patients who achieve hematuria remission by TSP than in those without hematuria remission [26]. Serum levels of galactose-deficient IgA1 and hematuria decrease in 60 % of all patients receiving tonsillectomy alone [27]. Moreover, adding steroid pulse therapy improves these parameters even when tonsillectomy alone has no apparent effect [27]. These findings suggest that tonsillectomy combined with steroid pulse therapy acts against the pathogenesis both in the tonsils and bone marrow. Deeper understanding of the relationship between tonsillar/mucosa disorders and the onset/progression of IgAN is required using the basic and clinical approaches.

This study has several limitations. The sample size was small, since this study targeted only patients with mild proteinuria (0.4–1.0 g/day) at diagnosis. Moreover, the number of patients, their background factors such as histological severity and treatment such as the numbers of courses of steroid pulse therapy and ratio of administration of RAS-I among three groups were not strictly equitable, because of the retrospective nature of this cohort study. There is possibility that more early stage of the patients in TSP group result in lower rate of administration of RAS-I than other groups, because concomitant use of RAS-I for the patients with normotensive and mild proteinuria might be overtreatment. We planned a multicenter study to increase the number of target patients, and controlled confounding factors using multivariate analysis. We did not evaluate histological severity using the Oxford classification, which is commonly used to evaluate specific lesions such as mesangial hypercellularity, endocapillary hypercellularity, segmental glomerulosclerosis, and tubular atrophy/interstitial fibrosis [28]. Instead, the grading system used herein was based on Japanese guidelines [18, 19], because entire histological severity needed to be regarded as a prognostic factor in this study design. We could not verify whether TSP therapy affected true renal outcomes such as ESKD, because renal function in most patients with early IgAN did not deteriorate during the follow-up period. Therefore, we mainly estimated the effects of treatment based on findings of proteinuria and hematuria. Some studies have shown a relationship between the control of proteinuria within normal levels and the long-term stabilization of renal function [13, 29, 30]. Nam et al. showed that eGFR declines more rapidly in patients with time-averaged proteinuria of 0.3–0.99 g/day than in those with time-averaged proteinuria of <0.3 g/day [30]. Indeed, the remission of proteinuria or CR could serve as a surrogate marker of stabilized renal function over the long-term. Criteria proposed in Japan during 2013 [31] define urinary remission as three consecutive negative findings over a six-month period of urinary sediment, red blood cell counts in <5/high power field and proteinuria of <0.3 g/day. We could not apply these criteria because this study was completed before these criteria were published.

In conclusion, TSP has potential for inducing CR in patients with IgAN and mild proteinuria. However, further studies of larger cohorts are needed to determine whether this treatment modality has a long-term benefit on renal survival. The verification of indications of the TSP therapy [32, 33] and relapse rates of urinary abnormalities after achieving CR [34] are also key issues to resolve before combination therapy can be established.
